# Maturation of West Nile Virus Modulates Sensitivity to Antibody-Mediated Neutralization

**DOI:** 10.1371/journal.ppat.1000060

**Published:** 2008-05-09

**Authors:** Steevenson Nelson, Christiane A. Jost, Qinq Xu, Jessica Ess, Julie E. Martin, Theodore Oliphant, Stephen S. Whitehead, Anna P. Durbin, Barney S. Graham, Michael S. Diamond, Theodore C. Pierson

**Affiliations:** 1 Viral Pathogenesis Section, Laboratory of Viral Diseases, National Institutes of Health, Bethesda, Maryland, United States of America; 2 Vaccine Research Center, National Institutes of Health, Bethesda, Maryland, United States of America; 3 Department of Medicine, Washington University School of Medicine, St. Louis, Missouri, United States of America; 4 Department of Molecular Microbiology, Washington University School of Medicine, St. Louis, Missouri, United States of America; 5 Department of Pathology & Immunology, Washington University School of Medicine, St. Louis, Missouri, United States of America; 6 Laboratory of Infectious Diseases, National Institutes of Health, Bethesda, Maryland, United States of America; 7 Center for Immunization Research, Department of International Health, Johns Hopkins Bloomberg School of Public Health, Baltimore, Maryland, United States of America; University of California Irvine, United States of America

## Abstract

West Nile virions incorporate 180 envelope (E) proteins that orchestrate the process of virus entry and are the primary target of neutralizing antibodies. The E proteins of newly synthesized West Nile virus (WNV) are organized into trimeric spikes composed of pre-membrane (prM) and E protein heterodimers. During egress, immature virions undergo a protease-mediated cleavage of prM that results in a reorganization of E protein into the pseudo-icosahedral arrangement characteristic of mature virions. While cleavage of prM is a required step in the virus life cycle, complete maturation is not required for infectivity and infectious virions may be heterogeneous with respect to the extent of prM cleavage. In this study, we demonstrate that virion maturation impacts the sensitivity of WNV to antibody-mediated neutralization. Complete maturation results in a significant reduction in sensitivity to neutralization by antibodies specific for poorly accessible epitopes that comprise a major component of the human antibody response following WNV infection or vaccination. This reduction in neutralization sensitivity reflects a decrease in the accessibility of epitopes on virions to levels that fall below a threshold required for neutralization. Thus, in addition to a role in facilitating viral entry, changes in E protein arrangement associated with maturation modulate neutralization sensitivity and introduce an additional layer of complexity into humoral immunity against WNV.

## Author Summary

West Nile virus (WNV) virions incorporate 180 envelope (E) proteins that are the primary target of neutralizing antibodies. As newly formed WNV virions are released from infected cells, the E proteins undergo a significant organizational change associated with maturation into an infectious virus. However, this process is not always efficient, as populations of infectious WNV include virions that did not complete the maturation process and may be heterogeneous with respect to the arrangement of E proteins on the virion. In this study, we found that neutralization by antibodies specific for epitopes commonly recognized in vivo is strongly impacted by the maturation state of WNV. Our studies suggest that maturation of WNV reduces the accessibility of some, but not all, epitopes on the virion for antibody binding. Virions that retain some immature character can be neutralized by monoclonal antibodies that fail to block infection of populations of WNV composed solely of mature virions. Similar results were found using polyclonal human serum obtained from volunteers of two clinical trials of candidate WNV vaccines. These studies identify unappreciated aspects of the antigenic complexity of WNV and highlight the importance of understanding the heterogenous forms of WNV that may be introduced into or replicating within the host.

## Introduction

*Flaviviruses* are a group of positive-stranded RNA viruses that are of global significance due to their widespread distribution and their ability to cause a variety of diseases in humans [1]. West Nile virus (WNV) is a mosquito-borne member of this genus and is the etiologic agent of West Nile encephalitis. WNV is endemic in parts of Africa, Australia, Europe, Asia, and the Middle East and has been responsible for periodic outbreaks of encephalitis in humans and horses. The introduction of WNV into North America in 1999 and its rapid spread across the United States into Canada, Mexico, and the Caribbean identifies this virus as an emerging pathogen of clinical and economic significance for the Western Hemisphere (reviewed in [2]). While seroprevalence studies indicate that most WNV infections of humans are subclinical, clinically apparent infections range from a febrile illness (West Nile fever) to more severe and potentially fatal neurologic disease [3]. Currently, no WNV vaccine has been approved for use in humans and treatment is supportive.

Flaviviruses are small (∼50 nm diameter) spherical virions composed of three structural proteins (envelope (E), premembrane (prM), and capsid (C)), a lipid envelope, and an ∼11 kilobase monocistronic RNA of positive-sense polarity [1]. Crystal structures of the E protein of several related flaviviruses (WNV, dengue virus (DENV), tick-borne encephalitis virus (TBE)) reveal an organization of three domains connected by flexible hinges (reviewed in [4]). Domain III (DIII) is an immunoglobulin-like fold that is thought to participate in interactions between virions and cellular factors associated with virus entry. Domain II (DII) is a long, finger-like domain that contains a stretch of 13 conserved, hydrophobic residues that form an internal fusion loop. DIII and DII are linked together by a β-barrel structure that comprises domain I (DI). The structure of prM, which forms heterodimers with the E protein during virion biogenesis, is presently unknown.

Flaviviruses assemble at the endoplasmic reticulum (ER) and bud into the lumen as immature virus particles [5]. Cryoelectron microscopic reconstructions of immature virions reveal an icosahedral arrangement of 60 trimeric spikes composed of prM∶E heterodimers in which the prM protein is positioned to cover the fusion peptide located at the distal end of each E protein of the trimer [6],[7]. In this position, prM may prevent conformational changes that would inactivate the E protein during virion egress through mildly acidic compartments of the secretory pathway [8],[9]. During transit through the *trans*-Golgi network (TGN), prM is cleaved by a cellular furin-like protease resulting in the formation of a small virion-associated M peptide and the release of the amino-terminal “pr” portion of the protein [10]. This required cleavage step promotes a rearrangement of E proteins on the surface of the virion and the formation of a mature virus particle. Mature flavivirus virions are relatively smooth and composed of 90 anti-parallel E protein dimers arranged with pseudo-icosahedral symmetry [11].

Antibodies are a critical component of host defenses against flavivirus infection and mediate protection via effector functions and by direct neutralization of virus (reviewed in [12]). The primary target for neutralizing antibodies is the E protein, although antibodies specific for prM have been identified [13]–[15]. More than twelve distinct epitopes have been identified on the surface of the E protein that elicit antibodies characterized by varying degrees of neutralization potency in vitro and efficacy in vivo [16]–[22]. Neutralization of flavivirus infection is a multiple “hit” phenomenon in which virus inactivation occurs once the number of antibodies bound to a virion exceeds a required threshold [23],[24]. Previous studies with an extremely potent neutralizing mAb specific for a highly accessible epitope on an upper lateral surface of WNV DIII (DIII-lr) suggest this threshold is approximately 30 mAbs [23].

The pseudo-icosahedral arrangement of E proteins on the virion displays the E protein in three distinct chemical environments defined by proximity to the two-, three-, or five-fold axes of symmetry [25]. Epitopes in each of these environments may be differentially accessible for antibody binding due to steric constraints imposed by adjacent E proteins on the virus particle [18], [26]–[28]. As a result, the number of sites available for binding may differ among structurally distinct epitopes on the virion. Antibodies that bind highly exposed determinants may exceed the stoichiometric threshold for neutralization by binding a small fraction of accessible epitopes on the virion (low occupancy). In contrast, epitopes predicted to be poorly exposed may require nearly complete occupancy to achieve threshold requirements for neutralization [23]. Furthermore, some epitopes on the virion may not be accessible to antibody engagement with a stoichiometry that exceeds the threshold required for neutralization. Thus, antibodies that recognize such epitopes may neutralize poorly, or not at all, even at concentrations that permit saturation because too few antibodies can simultaneously dock on the virion. Paradoxically, many antibodies that recognize poorly accessible epitopes on the mature virion still show neutralizing activity in vitro and in vivo [18],[26]. How antibodies engage poorly accessible epitopes on virions with a stoichiometry that permits neutralization is difficult to reconcile using existing static models of virion structure and envelope organization. In this study, we investigate how changes in flavivirus structure associated with virion maturation impact the neutralizing activity of antibodies to WNV.

## Results

### Neutralization of WNV by antibodies that recognize poorly accessible determinants

The neutralization potential of an antibody is governed by the number of sites on the virion available for binding (epitope accessibility) and the strength of binding (affinity) [23],[29],[30]. Although molecular modeling studies of the mature virion suggest that many of the known epitopes on the E protein are poorly accessible, antibodies to these determinants still neutralize infection to varying degrees [18],[26]. To investigate mechanisms that govern the potency of antibodies that target poorly accessible epitopes, high-resolution neutralization profiles were generated for a group of antibodies that recognize structurally distinct epitopes on the WNV E protein [17],[18] (Fig. S1). Neutralization potency was estimated for each antibody using a recently described and validated neutralization assay employing WNV reporter virus particles (RVPs) [23],[31]. RVPs are produced by complementation of a sub-genomic replicon by expression of the structural proteins of the virus *in trans*, allowing infection to be measured as a function of reporter gene expression [32].

**Figure 1:**
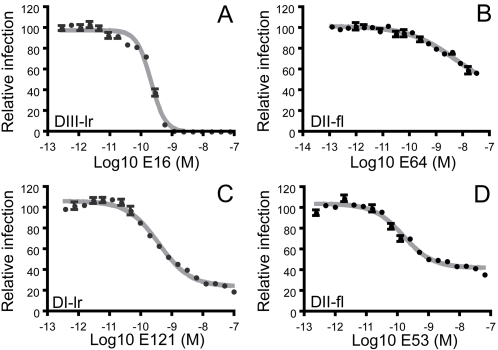
Dose-response profiles of DI/DII antibodies reveal a fraction of virions resistant to neutralization. Two-fold dilutions of mAbs E16 (A), E64 (B), E121 (C), and E53 (D) were incubated with WNV RVPs and used to infect Raji-DCSIGNR cells. The epitope recognized by each mAb was determined previously and is shown in each panel: DIII-lr (Domain III lateral ridge), DI-lr (Domain I lateral ridge), and DII-fl (Domain II fusion loop) [17],[18]. Error bars display the standard error of triplicate infections. Dose-response curves are representative of 2–6 independent assays.

Monoclonal antibodies (mAbs) that bind a highly accessible determinant on the upper lateral ridge of DIII (DIII-lr) potently neutralize WNV infection in vitro and exhibit significant protective capacity in vivo [16]–[18],[23],[31]. Dose-response curves obtained with the DIII-lr-specific mAb E16 were sigmoidal, relatively steep, and revealed complete neutralization of WNV RVPs at low concentrations of antibody (Fig. 1a; EC_50_ 0.043 nM) [23]. In contrast, none of the previously described E-protein-specific monoclonal antibodies recognizing determinants outside the DIII-lr were capable of completely blocking infection [18],[23] (Fig. 1b–d). By comparison with E16, dose-response profiles obtained for most DI- and DII-specific mAbs were relatively flat and revealed reduced neutralization potency, as illustrated by the DII fusion loop (DII-fl)-specific mAb E64 [Fig. 1b; EC_50_ 32.2 nM (n = 2)]. A second pattern was observed for mAbs E121 [Fig. 1c; EC_50_ 0.69 nM +/− 0.39 (n = 6)] and E53 [Fig. 1d; EC_50_ 1.0 nM +/− 1.4 (n = 5)] that recognize determinants on the lateral ridge of DI (DI-lr) and the DII-fl, respectively [18] (Fig. S1). Dose-response profiles for these antibodies were sigmoidal, but in contrast to results obtained with the DIII-lr specific mAb E16 (Fig. 1a), did not completely neutralize even at high concentrations of antibody. Instead, the level of neutralization plateaued to reveal a fraction of virions resistant to neutralization at any concentration of antibody tested. The existence of a fraction of virions resistant to neutralization even at saturating concentrations of antibody (∼32% +/− 9% and ∼19% +/− 7% for E53 and E121, respectively) implies heterogeneity in antibody accessibility among individual virions in the population, with some virions unable to accommodate antibody binding with a stoichiometry sufficient for neutralization [23].

### Manipulating the efficiency of WNV maturation

The arrangement of E proteins on the virion determines the accessibility of epitopes and thereby influences the neutralization potency of antibodies. Therefore, the dramatic changes in conformation and organization of E proteins on the virion during the flavivirus life cycle have the potential to impact antibody binding and function [6],[7],[25]. On immature virions, E proteins exist in trimeric spikes as heterodimers with the prM protein. The role of prM in this context is to regulate when E proteins acquire the capacity to mediate membrane fusion, presumably by controlling the oligomerization state of the E protein on the virion. Indeed, genetic studies indicate that furin-mediated cleavage of prM is required for the release of infectious virions [33]. However, relatively little is known about the relationship between the extent of prM cleavage and the acquisition of infectivity on individual virus particles. Virions may acquire the potential to infect cells following prM-cleavage-mediated activation of a relatively small fraction of the E proteins on the virion [8]–[10],[34],[35], as observed for the analogous E2 protein of alphaviruses [36]. Virions containing at least some uncleaved prM protein are present in bulk virus populations and are infectious [35],[37]. Because epitope accessibility may differ among individual viruses at intermediate or incomplete stages of maturation, we investigated the impact of virion maturation on the neutralization sensitivity of populations of virions containing different levels of uncleaved prM protein.

**Figure 2:**
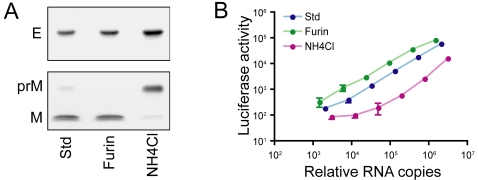
Manipulation of the maturation state of WNV RVPs. (A) RVPs incorporating different amounts of prM protein were produced by complementation of a BHK-21 cell line that stably propagates a WNV replicon as described in the Materials and Methods. The prM content of each population was analyzed by SDS-PAGE and Western blotting using E- and prM-specific mAbs. (B) The infectious titer of RVPs was measured by infecting Raji-DCSIGNR cells with serial four-fold dilutions of virus particles. Infection was measured 48 hours post-infection as a function of luciferase activity encoded by the replicon. The infectivity of each population of RVPs is shown relative to the genome content of the RVP preparation, which was measured using a modification of a previously described protocol [38]. Error bars indicate the standard error of triplicate infections. Data are representative of results obtained with 5 independently prepared stocks of RVPs.

WNV RVPs that are produced using our standard complementation conditions (std-RVPs) contain detectable amounts of uncleaved prM protein (Fig. 2a). This observation was true after production in several cell lines (BHK-21, 293T, Vero) (Fig. 2a, data not shown), and agrees with previous studies with infectious virus [9], [32], [38]–[40] or subviral particles [38]. Over-expression of human furin protease in transfected BHK-21 cells producing RVPs increased the efficiency of prM cleavage to levels that no longer allowed detection of unprocessed prM by Western blotting (furin-RVPs) [37],[41]. In contrast, treatment of BHK-21 producer cells with the weak base ammonium chloride reduced but did not absolutely block prM cleavage as has been described for other flaviviruses (NH_4_Cl-RVPs) [34]. Together, these modifications to our complementation approach allow for the production of RVPs that contain virtually no (furin-RVPs), low levels (std-RVPs), or high levels (NH_4_Cl-RVPs) of uncleaved prM. RVPs produced using all three transfection strategies are infectious, as shown by their capacity to infect Raji-DCSIGNR cells, albeit to differing degrees (Fig. 2b).

### Virus maturation reduces neutralization of WNV by anti-E antibodies in an epitope-specific manner

**Figure 3:**
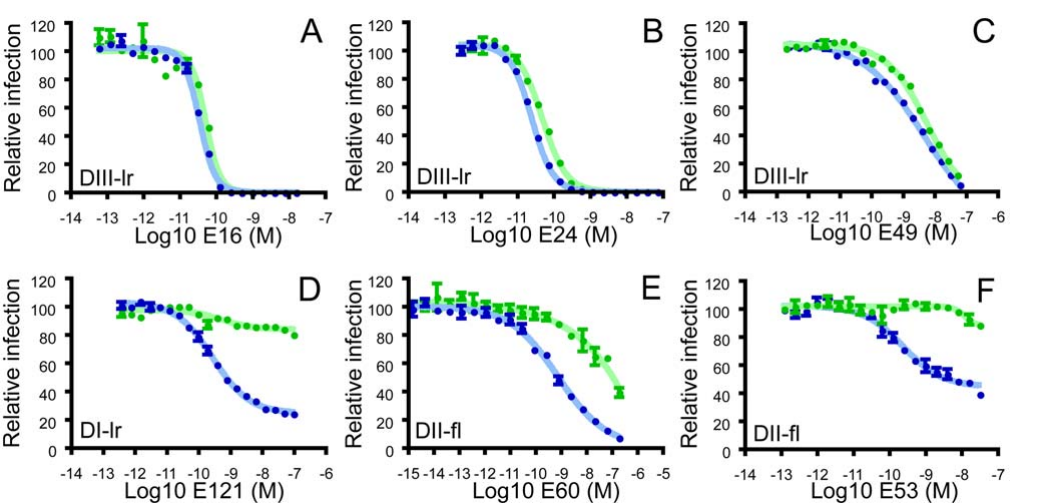
Maturation of WNV reduces sensitivity to neutralization by some but not all antibodies. The neutralization sensitivity of std- (blue symbols) and furin-RVPs (green symbols) was compared using mAbs that bind structurally distinct epitopes on the E protein. Two-fold dilutions of the DIII-specific mAbs E16 (A), E24 (B), E49 (C), the DI-specific mAb 121 (D), and the DII-specific mAbs E60 (E) and E53 (F) were incubated with WNV RVPs and used to infect Raji-DC-SIGNR cells. Error bars display the standard error of triplicate infections. Dose-response curves are representative of at least 3 independent assays.

To determine if the cleavage state of prM present on infectious virions impacts sensitivity to neutralization by anti-E antibodies, we compared the ability of mAbs to block infection of RVPs produced using either standard transfection conditions (Fig. 3; std-RVPs, blue circles) or cells that over-express human furin protease (Fig. 3; furin-RVPs, green circles). Three antibodies directed against the DIII-lr neutralized infection of both populations of RVPs with relatively similar potency (Fig. 3a–c; 0.9-, 1.5-, and 4.5-fold reduction in neutralization sensitivity of furin-RVPs for mAbs E16 (p = 0.67), E24 (p = 0.06), and E49 (p = 0.01), respectively). In contrast, mAbs recognizing determinants on the DI-lr (Fig. 3d, E121) and DII-fl (Fig. 3e, E60 and Fig. 3f, E53) displayed a markedly reduced capacity to neutralize infection of completely mature virions (furin-RVPs). When assayed using furin-RVPs, the potency of E60 was reduced approximately 95-fold relative to std-RVPs (4.3 +/− 2 nM (n = 8) vs. 408 +/− 113 nM (n = 8) for std- and furin-RVPs respectively (p<0.004)) whereas mAbs E53 (n = 5) and E121 (n = 6) did not neutralize a significant fraction of furin-RVPs.

**Figure 4:**
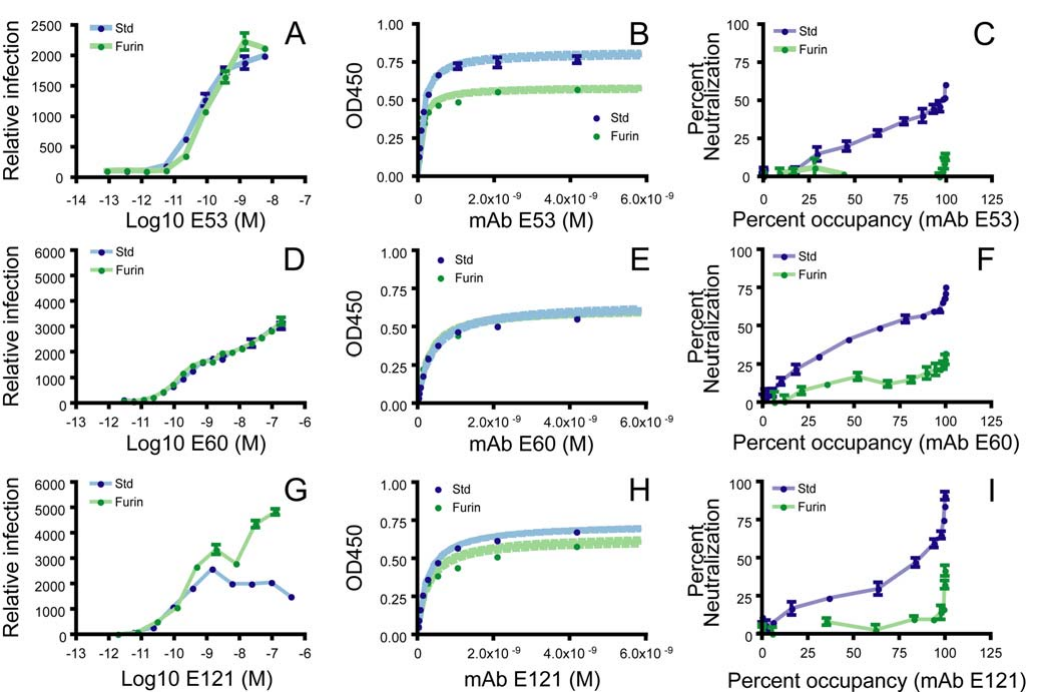
Neutralization by antibodies that target poorly accessible determinants occurs at complete occupancy of accessible epitopes. (A, D, G) Antibody-dependent enhancement of infection (ADE) describes an increase in the efficiency of infection of Fc-receptor bearing cells in the presence of non-neutralizing quantities of antibody. Std- (blue symbols) and furin-RVPs (green symbols) were mixed with serial four-fold dilutions of mAb E53 prior to infection of K562 cells. Infection was monitored as a function of GFP expression using flow cytometry. Infection efficiency is expressed relative to the percentage of K562 cells infected in the absence of antibody (set as 100). Error bars display the standard error of duplicate infections. (B, E, H) The affinity of mAb binding to RVPs was measured by ELISA using a particle-capture format. Data were analyzed as described in the Materials in Methods; error bars represent the standard errors of data from duplicate wells. The dotted line indicates the confidence interval for the regression analysis. (C, F, I) Occupancy requirements for neutralization were estimated by plotting data from mAb dose-response curves (y-axis) against the percentage of accessible epitopes bound by antibody at each point on the neutralization profile as described previously [23]. The percentage of accessible epitopes bound by antibody was computed using the affinity data obtained above by solving the equation: percentage bound = [Ab]/([Ab]+K_D_). Error bars display the standard error of triplicate infections.

The reduced ability of mAbs E53, E60, and E121 to neutralize mature virions was not due to an inability to bind the virus particle. Antibody-dependent enhancement of infection (ADE) describes the dramatic increase in infection of Fc-γ or complement receptor-bearing cells in the presence of sub-neutralizing concentrations of antibody or immune sera. Each of these mAbs strongly enhanced infection of std- and furin-RVPs in cells expressing activating Fc-γ receptors [42] (Fig. 4a, 4d, 4g, respectively). Consistent with this, both std-RVPs and furin-RVPs bound E53, E60, and E121 mAbs when either captured directly to plastic (data not shown), or used in a sandwich ELISA in which virus was captured using a humanized form of mAb E16 (a DIII-lr antibody that did not exhibit maturation-dependent differences in binding or neutralization). Using the latter approach, we measured the strength of binding of E53, E60, and E121 to WNV RVPs produced in the presence (furin-RVPs) or absence of furin (std-RVPs) (Fig. 4b, 4e, 4h, respectively). All three mAbs bound with relatively high affinity to both std-RVPs and furin-RVPs, with no significant differences in the strength of antibody binding to furin-RVPs relative to std-RVPs noted ((1.1 fold difference; p = 0.83), (1.2 fold difference; p = 0.67), and (1.3 fold difference; p = 0.38) for E53, E60, and E121, respectively).

The ability of the DI- and DII-specific mAbs to bind virions with relatively high affinity regardless of maturation state appears to conflict with their modest neutralizing capacity. Integration of affinity data with the neutralization dose-response curves, however, reveals that E53, E60, and E121 inhibit WNV infection only when a large fraction (99%) of accessible epitopes on virions are bound by antibody (Fig. 4c, 4f, 4i, respectively). The presence of a fraction of virions in std-RVP populations that remain infectious even at concentrations of antibody that allow complete occupancy suggests that a subset of virions do not display these epitopes at a stoichiometry sufficient to exceed the threshold required for neutralization [23]. In agreement, the occupancy requirements of these DI and DII mAbs suggest they bind an epitope that is not accessible on the average virion in std-RVP populations at a level that greatly exceeds the threshold requirements for neutralization [23]. Thus, even a small change in accessibility can significantly affect the potential for virion neutralization.

### Enhanced neutralization of WNV virions incorporating uncleaved prM

**Figure 5:**
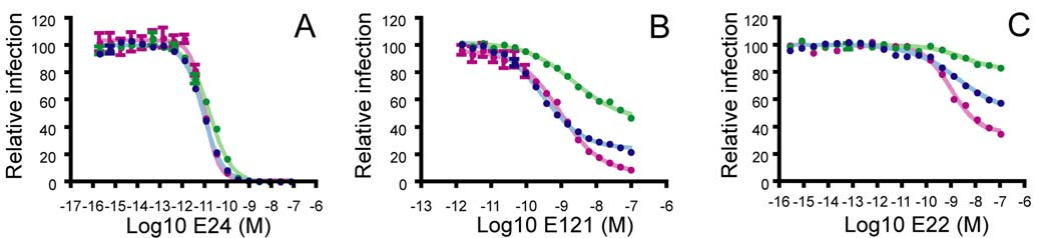
Neutralization sensitivity of RVPs that retain increased levels of prM. (A–C) The neutralization sensitivity of std- (blue symbols), furin- (green symbols), and NH_4_Cl-RVPs (purple symbols) was compared. Two-fold dilutions of the DIII-lr-specific mAb E24 (A), the DI-specific mAb 121 (B), and DIII-specific mAbs E22 (C) were incubated with WNV RVPs and used to infect Raji-DCSIGNR cells. Error bars display the standard error of triplicate infections. Three independent assays were performed.

If the changes in the arrangement of E proteins on virions that occur during maturation of WNV results in a reduction in the accessibility of epitopes in DI and DII, we would predict that populations of less mature virions containing greater amounts of non-cleaved prM should be more sensitive to neutralization by these mAbs. To test this hypothesis, WNV RVPs were produced in HEK-293T cells in the presence or absence of exogenous human furin plasmid, or in the presence of ammonium chloride, which inhibits prM cleavage. HEK-293T cells were used to produce the RVPs for these experiments because they support the release of higher titers of infectious RVPs relative to BHK-21 cells, allowing production of NH_4_Cl-RVPs at titers sufficient for neutralization studies under conditions of antibody excess. Neutralization profiles for all three populations of RVPs were generated using the indicated mAbs (Fig. 5). In agreement with experiments using BHK-derived RVPs, the potency of mAbs that recognize the highly accessible DIII-lr did not appreciably change when the proportion of mature and immature virus particles in the population was manipulated (Fig. 5a). In contrast, the DI- (E121; Fig. 5b) and DII-specific mAbs (E53; data not shown) were less effective at neutralizing furin-RVPs, but displayed an increased capacity to block infection of virions with increased levels of prM (NH_4_Cl-RVPs). Neutralization profiles by some DIII-specific mAbs exhibited a prominent resistant fraction, similar to our results with E121. E22 binds an epitope on a lower lateral surface of DIII that is poorly exposed on mature virions [23]. By comparison to studies with std-RVPs, dose-response studies with E22 revealed a reduced capacity to neutralize furin-RVPs, whereas studies with NH_4_Cl-RVPs revealed greater neutralizing activity (Fig. 5c). These results indicate that changes in neutralization sensitivity associated with maturation can occur for epitopes throughout the E protein and are not restricted to those in proximity to regions involved in prM-E protein interactions.

### Neutralization by polyclonal antibodies in vaccine recipients is sensitive to the state of WNV maturation

Recent studies indicate that the human humoral immune response to flavivirus infection is narrower than anticipated, with antibody specificity focused on determinants in the fusion loop in DII. B-cell repertoire analysis of three WNV-infected humans revealed that only 8% of WNV-specific B-cell clones produced antibodies specific to DIII, whereas almost half produced antibody that bound determinants in DII [43]. In addition, functional studies of the polyclonal response of WNV-infected horses and humans indicate that the neutralization activity of sera is not dependent upon antibodies directed against the DIII-lr epitope [44],[45]. Thus, during the natural course of flavivirus infection, many of the neutralizing antibodies present in the polyclonal response may be directed against determinants that are modulated by the maturation state of the virion.

**Figure 6:**
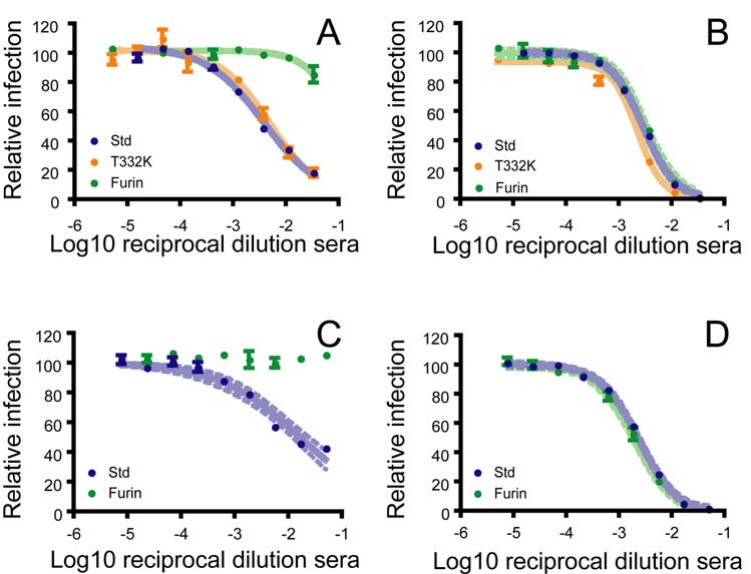
Polyclonal responses from vaccine recipients are sensitive to WNV maturation. (A–B) Neutralization profiles of the sera of twelve recipients twelve weeks after receiving a WNV DNA vaccine were obtained using furin-RVPs (green symbols) and a variant incorporating a single point mutation (T332K) that abrogates the binding of (and neutralization by) antibodies specific for the DIII-lr epitope (T332K: orange symbols) [17],[23]. Neutralization profiles with std-RVPs were obtained previously (blue symbols) [46]. Neutralization studies were performed on Raji-DCSIGNR cells with RVPs produced in BHK-21 cells. Neutralization potency of sera from two volunteers that were (A; volunteer G) or were not (B; volunteer D) sensitive to the maturation state of WNV are displayed for all three types of RVPs. (C–D) Neutralization profiles of the sera of six recipients of a live-attenuated WNV vaccine were analyzed using std- and furin-RVPs. Neutralization potency of serum from two volunteers that differ significantly in their capacity to neutralize furin-RVPs are shown (compare panels C (volunteer FF) and D (volunteer II)). Data for all vaccinated subjects are displayed in Fig. S2.

To investigate whether the potency of a polyclonal immune response is sensitive to the maturation state of WNV, we obtained serum from participants of two different phase I clinical trials of candidate WNV vaccines. First, the neutralizing antibody response in serum of twelve individuals immunized with a DNA vaccine encoding WNV prM-E was characterized [46],[47]. This vaccine encodes the prM-E proteins of WNV, and is thought to promote the release of subviral particles in vivo. RVPs that incorporate a single amino acid substitution in the E protein (T332K) that abrogates antibody binding to all strongly neutralizing mAbs that map to the DIII-lr epitope [16],[17],[23] were used to establish that the neutralizing response to this vaccine was associated with non-DIII-lr antibodies. Neutralization titers obtained with T332K-RVPs were not significantly reduced in any individual when compared to std-RVPs (Fig. S2a), consistent with prior studies in WNV infected humans and horses [44],[45]. In contrast, in parallel studies performed with furin-RVPs, we observed a significant reduction in the capacity of sera from half of the recipients to neutralize furin-RVPs as compared to std-RVPs (Fig. 6a and S2b). In fact, some individuals mount a response with very little capacity to neutralize mature virus (furin-RVPs) (compare Fig. 6a and 6b).

Because the E proteins of subviral particles are arranged with a different geometry (T = 1) relative to the pseudo-icosahedral (T = 3) structure of infectious virions, it is possible that the repertoire of antibodies elicited by this form of vaccination may be more sensitive to the maturation state of WNV than one elicited by an infectious virion. Therefore, we also characterized the polyclonal response of six recipients of a live-attenuated WNV vaccine. In agreement with the results described above, the neutralizing antibody response of roughly half the recipients of the live-attenuated candidate vaccine also displayed a significantly reduced capacity to neutralize furin-RVPs relative to their potency against std-RVPs (Fig. S2c). Two of these subjects failed to neutralize furin-RVPs at all, despite a capacity to neutralize std-RVPs (compare Fig. 6c and 6d). In contrast, sera from two of these recipients neutralized furin-RVPs at modestly higher titers than observed with std-RVPs. As the concentrations and specificities of the individual antibodies that comprise a polyclonal response are probably dynamic and could differ following vaccination with different antigens or using different immunization schedules, additional studies will be required to understand the factors that determine the sensitivity of the humoral response to heterogenous populations of flaviviruses. Together, these studies suggest that the maturation state of WNV represents an additional layer of antigenic complexity, with the polyclonal response of some vaccine recipients directed against epitopes with a reduced capacity to neutralize mature virions.

## Discussion

Neutralization of WNV is a “multiple-hit” phenomenon that requires the simultaneous engagement of the virus particle by as many as 30 antibody molecules [23],[24]. In this regard, the neutralization potential of an antibody is determined by the strength of binding and the abundance of its epitope on the virion (reviewed in [29]). Antibodies that bind the highly accessible DIII-lr epitope can exceed the threshold required for neutralization by binding a fraction of available epitopes displayed on the virion [23]. However, many of the epitopes recognized by antibodies produced following natural infection are not predicted to be accessible on the fully mature virion due to steric constraints imposed by the pseudo-icosahedral arrangement and packing of the E protein [18],[26]. Antibodies that recognize poorly exposed epitopes must bind a larger fraction of sites to exceed the stoichiometric requirements for neutralization [23]. Consistent with this, studies with mAbs E53, E60, and E121 which bind two distinct, poorly exposed epitopes on the E protein (DII-fl and DI-lr) indicate that neutralization of WNV infection occurs only when all (>99%) accessible determinants on the average virion are engaged by antibody.

For antibodies that bind epitopes requiring virtually complete occupancy to achieve neutralization, even modest changes in epitope accessibility can significantly affect the outcome of antibody binding. While the changes in E protein organization that define flavivirus maturation provide a mechanism for regulating the fusion activity of the class II glycoproteins of the virion [48], a consequence of maturation may be to mask epitopes recognized by the humoral response. Increasing the efficiency of prM cleavage (virion maturation) significantly reduces neutralization potency of many antibodies specific for epitopes predicted to be poorly exposed on the mature virion. This is apparent by comparing the neutralization profiles of the DI-specific mAb E121 with RVPs containing virtually no (furin-RVPs), low levels (std-RVPs), or high levels (NH_4_Cl-RVPs) of uncleaved prM. In contrast, potently neutralizing mAbs specific for a highly accessible epitope on the DIII-lr (e.g., E16) neutralize WNV RVPs regardless of the amount of prM present. Antibodies specific for poorly accessible epitopes do not completely lose the capacity to bind mature virions, nor do they bind with significantly reduced affinity. Instead, our studies suggest that maturation reduces the number of antibodies that may simultaneously bind the virion to levels that do not exceed a required threshold for neutralization even at full occupancy.

Because the arrangement of E protein on “partially mature” virions that retain uncleaved prM protein has not been resolved by structural studies, it remains uncertain how prM increases the accessibility of epitopes that are poorly accessible on the mature virion. While not observed in our studies, the presence of prM on virions will likely reduce the binding of some epitopes, as has been observed by ELISA using DENV and TBE [8],[35]. However, biochemical analyses of bulk virion populations may be limited, as these methods average the contribution of individual virus particles without regard to their infectious potential. While none of the antibodies analyzed in this study bind prM directly, one potential mechanism for an increased stoichiometry of binding to prM-containing viruses is an increase in the accessibility of epitopes on the E proteins when arranged as trimers associated with prM, relative to their accessibility in the pseudo-icosahedral mature virion. However, several of the mAbs with altered neutralization sensitivity recognize the fusion loop, which may be masked by direct interactions with prM [7]. Alternatively, changes in E protein epitope accessibility associated with maturation may reflect dynamic aspects of virion structure that are not evident from existing structural studies. Cryo-EM reconstructions provide an average structural state of E proteins on the virion. These static models of E protein arrangement cannot account for antibody binding to transiently exposed determinants that result from dynamic and/or lateral movement among E proteins on the virion. Partially mature virions that contain uncleaved prM may be more dynamic than mature virus with respect to the number or stability of alternate structural conformations that E proteins can attain.

Depending upon the proportion of immature, partially mature, and mature virions in a population of WNV, the same antibody may have little, modest, or significant neutralizing activity. From a technical perspective, maturation state-dependent neutralization has significant implications for the reproducibility of neutralization tests among laboratories, and may be important for determining whether antibodies in a given serum sample are judged as protective. More importantly, these studies suggest that the protective capacity of an antibody may depend in part upon the maturation state of virus delivered through the bite of a mosquito or released from infected human tissues in vivo. While the functional contribution of antibodies of different specificities in sera from infected or vaccinated humans is not yet understood, humans do produce large numbers of antibodies specific for determinants outside of the DIII-lr [43],[44]. These may exhibit a significantly reduced potential to neutralize mature WNV. Indeed, half the recipients of two candidate WNV DNA vaccines evaluated in this study generated a polyclonal response that was notably less effective at neutralizing mature virus. Whether response to immunization with other classes of WNV vaccines, different immunization schedules, or natural infection will result in responses more capable of neutralizing all forms of the virus is of significant interest. Together these studies suggest that a consequence of maturation of WNV is a reduction in sensitivity to neutralization by antibodies recognizing specificities that are an important component of the humoral response to infection and vaccination. The influence of maturation on the neutralization sensitivity of WNV identifies an unappreciated functional consequence for the heterogeneity of prM cleavage in populations of flaviviruses [8]–[10],[34],[35],[49], and introduces an additional layer of complexity into analyses of humoral immunity against WNV.

## Materials and Methods

### Cell lines

BHK-21 WNIIrep-G/Z, 293T, Vero, Raji-DCSIGNR and K562 cells were maintained as described previously [23],[31].

### WNV immune sera

Neutralization studies were performed on sera obtained from two WNV vaccine trials sponsored by the NIH. First, sera were obtained from twelve participants in a single-site, Phase I, open-label study to examine the safety, tolerability, and immune response to an investigational recombinant DNA WNV vaccine encoding prM and E, described in detail elsewhere [46]. Second, sera from six participants of a Phase I double-blinded, placebo-controlled study to evaluate the safety, infectivity, attenuation and immunogenicity of a live-attenuated WNV/DENV4 vaccine were obtained for analysis (A. Durbin, unpublished). These studies and subsequent analyses were performed in compliance with the guidelines of The U.S. Department of Health and Human Services (DHHS), and the protocols were approved by the respective Institutional Review Boards. Neutralization studies with WNV-immune sera were performed on Raji-DCSIGNR cells with RVPs produced in BHK-21 cells as described below.

### Production of WNV RVPs

RVPs were produced using methods and constructs described previously [23],[31],[41]. For RVPs produced in BHK cells, BHK-21 WNIIrep-G/Z cells were transfected with a plasmid encoding WNV C-prM-E and an empty pcDNA vector using a 1∶3 ratio by mass (std-RVPs). Std-RVP stocks derived from HEK-293T cells were produced by transfection with plasmids encoding either the WNIIrep-G/Z or WNIIrep-REN replicon, C-prM-E, and pcDNA using a 0.5∶1∶2.5 ratio. Maturation of RVPs produced in either cell type was enhanced (furin-RVPs) by including a plasmid encoding human furin protease in place of the pcDNA vector. To produce immature RVPs, media from transfected cells was exchanged with media containing 20 mM ammonium chloride (in phosphate buffered saline) at 12 hours post-transfection, and again two hours later. Transfections were performed in T75 flasks using 40 µg of DNA and Lipofectamine 2000 (Invitrogen, Carlsbad, CA) and harvested at 48 hours post-transfection. The efficiency of prM cleavage of populations of RVPs produced in either BHK-21 or HEK-293T cells was determined by Western blotting as described below.

### Measuring the infectious titer of WNV RVPs

The infectious titer of RVP preparations was determined by infecting Raji-DCSIGNR cells with serial four-fold dilutions of RVPs in triplicate. Cells were lysed 36–40 hours post-infection and assayed for luciferase activity according to the manufacturer's instructions (Promega, Madison, WI). To relate the infectivity of each population of RVPs relative to the number of particles in the supernatant, the RNA content of each stock was measured as described previously [38].

### Neutralization and enhancement of WNV RVP infection

Neutralization studies were performed using Raji-DCSIGNR cells as described previously [18],[23]. WNV RVP stocks were diluted and incubated with mAb for 60–120 minutes at room temperature. Antibody-RVP complexes were then added to pre-plated cells in triplicate. Percent infection was measured by flow cytometry at 48 hours after RVP addition. The EC_50_ of each antibody was predicted by non-linear regression analysis using a variable slope. Statistical comparisons of the neutralization potency of different mAbs were performed using the T-test (GraphPad Prism 4, GraphPad Software Inc., San Diego CA). Antibody-dependent enhancement of infection was measured using K562 cells that express the activating Fc-γ receptor CD32a as described [23].

### Detection of prM in WNV RVPs by Western blot analysis

RVPs were harvested at 48 hours post-transfection, filtered using a 0.22 µM filter, and concentrated and partially purified by ultracentrifugation through a 20% sucrose cushion. RVPs were resuspended in TNE buffer (100 mM Tris, 2 M NaCl, 100 mM EDTA, pH adjusted to 7.4) and analyzed using SDS-PAGE and Western blotting. E protein was detected using the mAb E16 (1 µg/ml), whereas prM was detected using a commercial antibody specific for residues 8–27 of the WNV M protein (1 µg/ml) (Imgenex, San Diego, CA).

### Antibody affinity measurements

Antibody affinity was measured using an indirect ELISA and WNV RVPs produced in HEK-293T cells. High-protein-binding plates were coated with a 1 µg/ml humanized mAb E16 overnight at 4 °C using alkaline conditions. Plates were blocked in blocking buffer (BB: 1X PBS, 0.05% Tween-20, and 1% BSA) and WNV RVPs diluted in BB were then bound at room temperature for 2 hours with gentle shaking. Virus particle-coated plates were then incubated in the presence of serial dilutions of anti-WNV antibodies under conditions of antibody excess. Bound antibody was detected using a horseradish peroxidase-conjugated goat anti-murine kappa chain antibody. Antibody affinity was estimated using non-linear regression with a hyperbolic equation that describes the binding of a ligand to a receptor under conditions that follow the law of mass action (one-site binding equation; Bound = ((Bmax)(X)/(K_D_+X)). Statistical comparisons were made using the T-test (GraphPad Prism 4).

The occupancy requirements for neutralization by each antibody were estimated by plotting data from mAb dose-response curves (y-axis) against the percentage of accessible epitopes bound by antibody at each concentration of antibody as described previously [23]. The percentage of accessible epitopes bound by antibody was computed at each point on the dose-response curve using the affinity data obtained above by solving the equation: percent bound = [Ab]/([Ab]+K_D_).

## Supporting Information

Figure S1 Maturation of WNV reduces sensitivity to neutralization by some but not all antibodies. Ribbon diagram of the WNV E protein highlighting residues that form the epitopes recognized by mAbs used in this study. Domains II, I, and III are shown as yellow, red, and blue ribbons. Residues on the Domain III lateral ridge (DIII-lr) involved in recognition by mAbs E16, E24, and E49 are indicated as orange spheres. Epitopes recognized by E121 (Domain I lateral ridge: DI-lr), E53, and E60 (both in the Domain II fusion loop: DII-fl) are identified as yellow, blue, and red spheres, respectively. Overlapping residues recognized by both E60 and E53 are shown in purple.
(0.47 MB TIF)

Figure S2 Polyclonal responses from vaccine recipients are sensitive to WNV maturation. Neutralization profiles of sera from twelve recipients of a WNV DNA vaccine twelve weeks post-vaccination were obtained using RVPs incorporating the T332K mutation (A) and furin-RVPs (B). These studies were performed on Raji-DCSIGNR cells with RVPs produced in BHK-21 cells. Dose-response curves were obtained and analyzed as described above using serial three-fold dilutions of sera. The EC_50_ obtained with std-, T332K- and furin-RVPs for all volunteers studied is displayed with error bars indicating the standard error obtained using 2–4 independent assays. (C) Neutralization profiles of sera from six recipients of a single dose, live-attenuated WNV vaccine six weeks post-vaccination were obtained using furin-RVPs as described above. The average EC_50_ obtained is displayed, with error bars indicating the standard error of 3 independent assays. * = p<0.05, ** = p<0.01, *** = p<0.005.
(0.26 MB TIF)
