# Supplementary figures and images for: Maturation of West Nile Virus Modulates Sensitivity to Antibody-Mediated Neutralization
Source: PLoS Pathog. 2008 May 9;4(5):e1000060. doi: 10.1371/journal.ppat.1000060 (PMC2330159; doi:10.1371/journal.ppat.1000060)

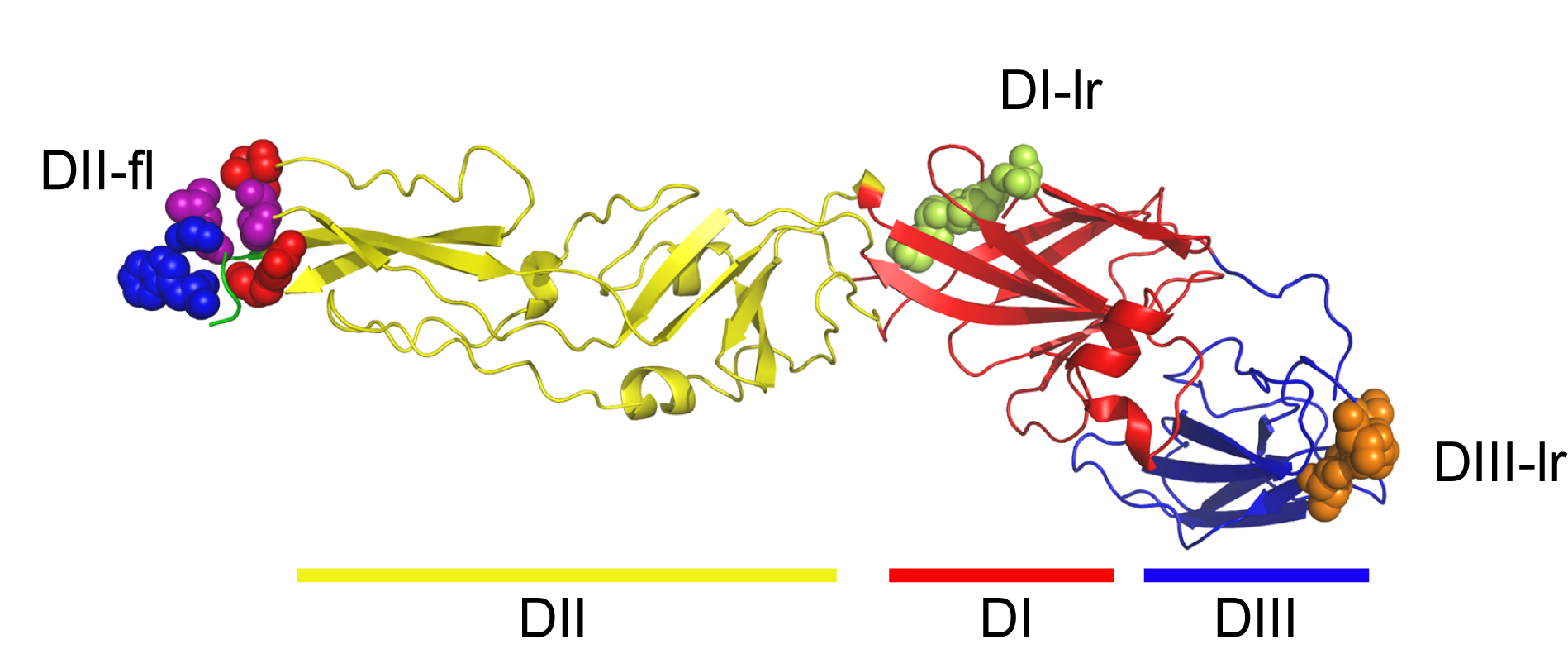

Supplement: Figure S1 — Maturation of WNV reduces sensitivity to neutralization by some but not all antibodies. Ribbon diagram of the WNV E protein highlighting residues that form the epitopes recognized by mAbs used in this study. Domains II, I, and III are shown as yellow, red, and blue ribbons. Residues on the Domain III lateral ridge (DIII-lr) involved in recognition by mAbs E16, E24, and E49 are indicated as orange spheres. Epitopes recognized by E121 (Domain I lateral ridge: DI-lr), E53, and E60 (both in the Domain II fusion loop: DII-fl) are identified as yellow, blue, and red spheres, respectively. Overlapping residues recognized by both E60 and E53 are shown in purple. (0.47 MB TIF) [file ppat.1000060.s001.tif]

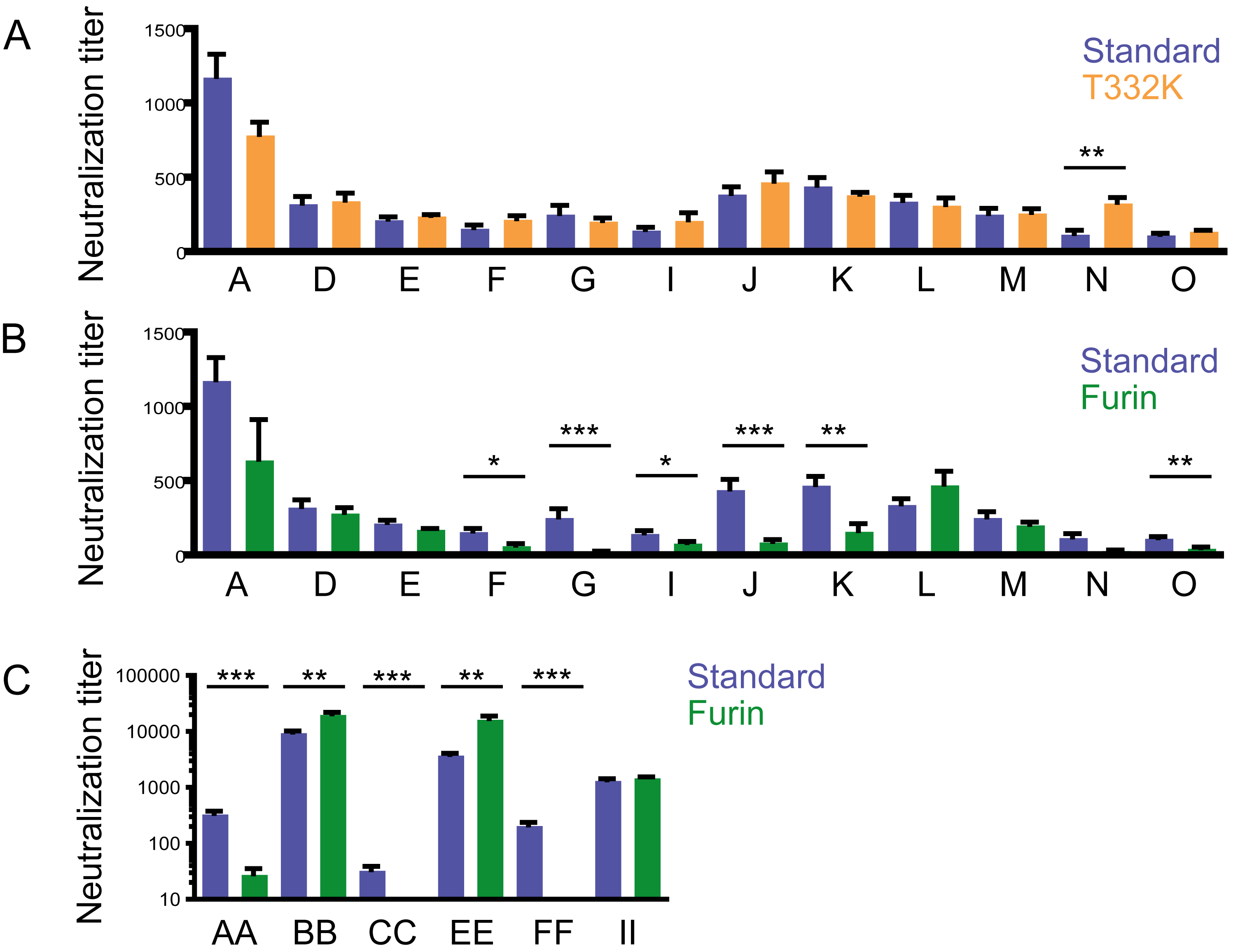

Supplement: Figure S2 — Polyclonal responses from vaccine recipients are sensitive to WNV maturation. Neutralization profiles of sera from twelve recipients of a WNV DNA vaccine twelve weeks post-vaccination were obtained using RVPs incorporating the T332K mutation (A) and furin-RVPs (B). These studies were performed on Raji-DCSIGNR cells with RVPs produced in BHK-21 cells. Dose-response curves were obtained and analyzed as described above using serial three-fold dilutions of sera. The EC50 obtained with std-, T332K- and furin-RVPs for all volunteers studied is displayed with error bars indicating the standard error obtained using 2–4 independent assays. (C) Neutralization profiles of sera from six recipients of a single dose, live-attenuated WNV vaccine six weeks post-vaccination were obtained using furin-RVPs as described above. The average EC50 obtained is displayed, with error bars indicating the standard error of 3 independent assays. * = p<0.05, ** = p<0.01, *** = p<0.005. (0.26 MB TIF) [file ppat.1000060.s002.tif]
